# House and Office Heating

**Published:** 1906-06-16

**Authors:** 


### HOUSE AND OFFICE HEATING.

### TESTS OF GRATES FOR DOMESTIC USE.

The Coal Smoke Abatement Society recently issued an important report, giving the results of a series of tests of grates lately carried out by that Society, under the auspices of the War Office. Some thirty-six grates were tested in two preliminary tests, and five open grates which gave the best results were submitted to further final and exhaustive experiments. The five grates selected by the examiners to be finally tested were those of Messrs. Candy and Company, J. and R. Corker, Hendry and Pattison (Boyd's), the London Warming and Ventilating Company, and the Teale Fireplace Company. On the suggestion of Mr. A. C. Ackerman, M.I.C.E., the grates in the final series were submitted to the carbon-dioxide test, with the object of ascertaining their efficiency as coal-burning machines for producing heat. For this test the sub-committee secured the services of Dr. J. S. Owens, A.M.I.C.E., who reported in effect that in considering the two best grates—D. O. Boyd's improved hygiastic grate (Hendry and Pattison) and Candy and Company's—the quantity of heat given to the walls, chiefly by radiation, is very great, amounting to over 79 per cent. in the former and to 76 per cent. in the latter; but it must not be thought that this is all lost heat. It is well known that we feel warm in a room with warm walls in which the air is cold, and that we feel cold in a room with cold walls in which the air may be warm. This is largely because air is such a bad conductor of heat and because we are very sensitive to radiant heat. It appears from certain very complete tests of a hot-water warming plant at a large London hospital, made by Mr. Ackerman, that 22 per cent. of the heat discharged from the radiators was utilised in warming the air of the room, whilst the remaining 78 per cent. was radiated into the walls and through the windows. It will thus be seen that the percentage of heat taken by the walls was practically the same for the radiators and open fires, while the efficiency of the radiators in warming the air of the room was almost three times as great as that of the open fires. If, however, we consider the efficiency of a hot-water plant, taking into account all heat losses from the furnace to the radiators, the figures become more alike, being 8 per cent. of the heat of the fuel burnt for the hot-water plant, as against 7 per cent. for the open fires.

In the case of the open fires the chimney losses (14 to 16 per cent.) are about twice as great as compared with the efficiency. In the case of the radiators there are no chimney losses, the rooms being the equivalent of the chimneys and rooms together in the case of the open fires. If for the open fires (the hygiastic grate and Candy and Company's) we add the chimney losses to the heat given to the air of the room we have, say, 21 per cent. and 23 per cent. as the combined efficiency, which compare very well with the 22 per cent. which Mr. Ackerman obtained in the case of radiators.

It is reported that the meteorological conditions during the period of testing were highly favourable; that all these grates were worked with the object of attaining their utmost capacity, and not under the conditions obtaining in an ordinary room, which would generally be more valuable. The fires were not allowed to burn low, so that the amount of smoke emitted in these tests was the minimum that could be expected.

In the result the examiners found that of the grates submitted to them those of Messrs. J. and R. Corker, Messrs. Candy and Company, and Messrs. Hendry and Pattison (Boyd's) are the best, showing practically equal results, and that the "Florence" (the London Warming and Ventilating Company) very nearly approximates to them.

